# Multi-epitope chimeric antigen used as a serological marker to estimate *Plasmodium falciparum* transmission intensity in the border area of China-Myanmar

**DOI:** 10.1186/s40249-016-0194-x

**Published:** 2016-09-07

**Authors:** Mei-Xue Yao, Xiao-Dong Sun, Yu-Hui Gao, Zhi-Bin Cheng, Wei-Wei Deng, Jia-Jia Zhang, Heng Wang

**Affiliations:** 1 Department of Microbiology and Parasitology, Institute of Basic Medical Sciences, Chinese Academy of Medical Sciences and School of Basic Medicine, Peking Union Medical College, Beijing, 100005 China; 2 Yunnan Institute of Parasitic Diseases, Puer, Yunnan China; 3 Department of Biochemistry and Molecular Biology, Institute of Basic Medical Sciences, Chinese Academy of Medical Sciences and School of Basic Medicine, Peking Union Medical College, Beijing, China

**Keywords:** *Plasmodium falciparum*, Serology, Multi-epitope chimeric antigens, Transmission intensity, China-Myanmar border

## Abstract

**Background:**

Following the decline of malaria transmission in many countries and regions, serological parameters have become particularly useful for estimating malaria transmission in low-intensity areas. This study evaluated a novel serological marker, Malaria Random Constructed Antigen-1 (M.RCAg-1), which contains 11 epitopes from eight *Plasmodium falciparum* antigens, as a tool for assessing malaria transmission intensity along the border area of China-Myanmar.

**Method:**

Serum from *Plasmodium falciparum* and *P. vivax* patients was used to detect the properties of M.RCAg-1 and antibody responses. Cross-sectional surveys were conducted at the China-Myanmar border and in Hainan province in 2012 and 2013 using cluster sampling. Filter blood spot papers were collected from all participants. Antibodies against M.RCAg-1 were detected using indirect ELISA. The Mann–Whitney test and Spearman’s rank correlation test were performed to analyze antibody data. *P. falciparum* malaria transmission intensity was estimated using a catalytic conversion model based on the maximum likelihood of generating a community seroconversion rate (SCR).

**Results:**

M.RCAg-1 was well-recognized by the naturally acquired anti-malaria antibodies in *P. falciparum* patients and had very limited cross-reactivity with *P. vivax* infection. The total amount of IgG antibodies was decreased with the decrease in parasitemia after taking medication and lasted several weeks. In a population survey, the antibody levels were higher in residents living close to the China-Myanmar border than those living in non-epidemic areas (*P* < 0.0001), but no significant difference was observed between residents from Hainan and non-epidemic areas. The calculated SCR was 0.0128 for Jieyangka, 0.004 for Susuzhai, 0.0047 for Qiushan, and 0.043 for Kayahe. The estimated exposure rate obtained from the anti-M.RCAg-1 antibody level correlated with traditional measures of transmission intensity derived from altitude.

**Conclusion:**

Our study demonstrates that M.RCAg-1 is potentially useful as a serological indicator of exposure to *P. falciparum* malaria, especially for malaria surveillance in low transmission areas.

**Electronic supplementary material:**

The online version of this article (doi:10.1186/s40249-016-0194-x) contains supplementary material, which is available to authorized users.

## Multilingual abstracts

Please see Additional file 1 for translations of the abstract into the five official working languages of the United Nations.

## Background

Approximately 3.3 billion people are still at risk for malaria globally, most of them infected by *Plasmodium falciparum* (*Pf*) [1], even though the malaria burden has decreased after a long-term effort implemented in endemic areas. In recent years, many countries and regions that were threatened by malaria previously have entered a malaria-eliminating stage [2]. Unlike malaria control, malaria elimination needs to interrupt local mosquito-borne malaria transmission within a defined geographical area with no incidence of locally contracted cases [3], which makes effective surveillance and transmission intensity tracing necessary at this stage. Accurate assessment of the *Pf* transmission intensity can help us understand the disease burden and the risk of being infected, provide guidance for control and prevention strategies and confirm an area is *Pf* malaria-free with reliable evidence. Until now, the industry standard for malaria transmission intensity has been entomological inoculation rate (EIR), which is time-consuming, expensive, and imprecise in low-transmission districts [4]. Other methods, such as climate-based models, parasite prevalence, and mean hemoglobin concentration, indirectly reflect *Pf* transmission intensity. However, these methods are not sensitive enough in communities and are inaccurate, especially in areas of low transmission.

To efficiently assess malaria transmission and endemicity, many researchers have suggested that serological parameters offer more advantages than other approaches, such as EIR [5–8], because antibodies depend on exposure to malaria infection and can persist for a long time. Therefore, this tool could allow us to detect changes in malaria transmission over time and monitor the effectiveness of malaria control programs. However, whether an appropriate serological marker can be selected is the critical core of this method. Various malaria antigens have been used as serological markers [9, 10], including apical membrane antigen-1 (AMA-1), merozoite surface protein-2 (MSP-2), and merozoite surface protein-1_19_ (MSP-1_19_). Antibodies elicited by these well-characterized *Pf* antigens have been tested at relatively stable rates in some communities. However, each of the individual antigens has its own limitations. For example, MSP-2 with a high rate of polymorphism [11, 12] will underestimate the seroprevalence with population differences [13]; saturation of antibody prevalence is easy to achieve with AMA-1 with high immunogenicity [14], even at moderate malaria endemicity, which makes them effective in areas of extremely low endemicity or for determining the extent of malaria epidemics [15]; and MSP-1_19_ has been used to estimate *Pf* malaria transmission in many African areas [16], but the antibodies to this antigen can persist for years, with a half-life of nearly 50 years, so this sluggish response to changes make it inappropriate for assessing deviations in *Pf* transmission in the short-term. Considering of great individual variations in antibody responses and multiple malaria antigens expressed during the process of *Pf* infection [17], antibody responses to single antigens are circumscribed and inadequate as biomarkers for indicating malaria transmission intensity [18]. A multiplex assay based on Luminex technology, which can detect multiple antigens simultaneously, have been developed and used for a long time [19–22]. However, the high investment costs and complex operations may prevent it from being widely used.

More novel serological biomarkers are being found that can accurately estimate recent *Pf* exposure for not only communities [23], but also individuals. However, the detection of antibodies to several antigens is also a relatively big job, and the polymorphic reaction to these natural antigens of *Pf* in different populations is still difficult to avoid. Our lab has successfully constructed a multi-epitope chimeric protein, Malaria Random Constructed Antigen-1 (M.RCAg-1) [24], which contains 11 relatively conservative epitopes from eight *Pf* antigens. This chimeric antigen was selected from a DNA library containing thousands of diverse multi-epitope chimeric antigen genes constructed using epitope shuffling and an isocaudamer technique due to its high specific immunogenicity and anti-parasite efficacy [25].

The objective of this study was to estimate whether M.RCAg-1 can be used as an indicator of *Pf* malaria transmission dynamics. Using a simple indirect ELISA, we detected anti-M.RCAg-1 antibodies in the serum of *Pf*-infected patients from *Pf* malaria endemic areas or non-endemic areas. The serological parameter obtained in our study demonstrated that this chimeric antigen can be used as an indicator to estimate malaria transmission dynamics.

## Methods

### Malaria patients

Serum samples were collected from malaria patients in Laza, Myanmar, between September and December 2008 to detect antibody responses against M.RCAg-1. The patients were diagnosed by microscopic examination of Giemsa-stained blood smears and treated promptly with the appropriate antimalarial and supportive therapy. Four plasma samples were collected from each patient: prior to drug therapy (D0), the first day of treatment (D1), the third day after treatment (D3), and the seventh day after treatment (D7). A Giemsa-stained blood smear was prepared at the same time plasma samples were collected. Plasma was used for the detection of antibody against M.RCAg-1 and epitopes. Giemsa-stained blood smears were used to determine parasitemia levels and the Plasmodium species. A total of 67 *Pf* patients and 38 *Plasmodium vivax* (*Pv*) patients were enrolled in the study. The population details have been described elsewhere [26].

### Study sites

**Fig. 1:**
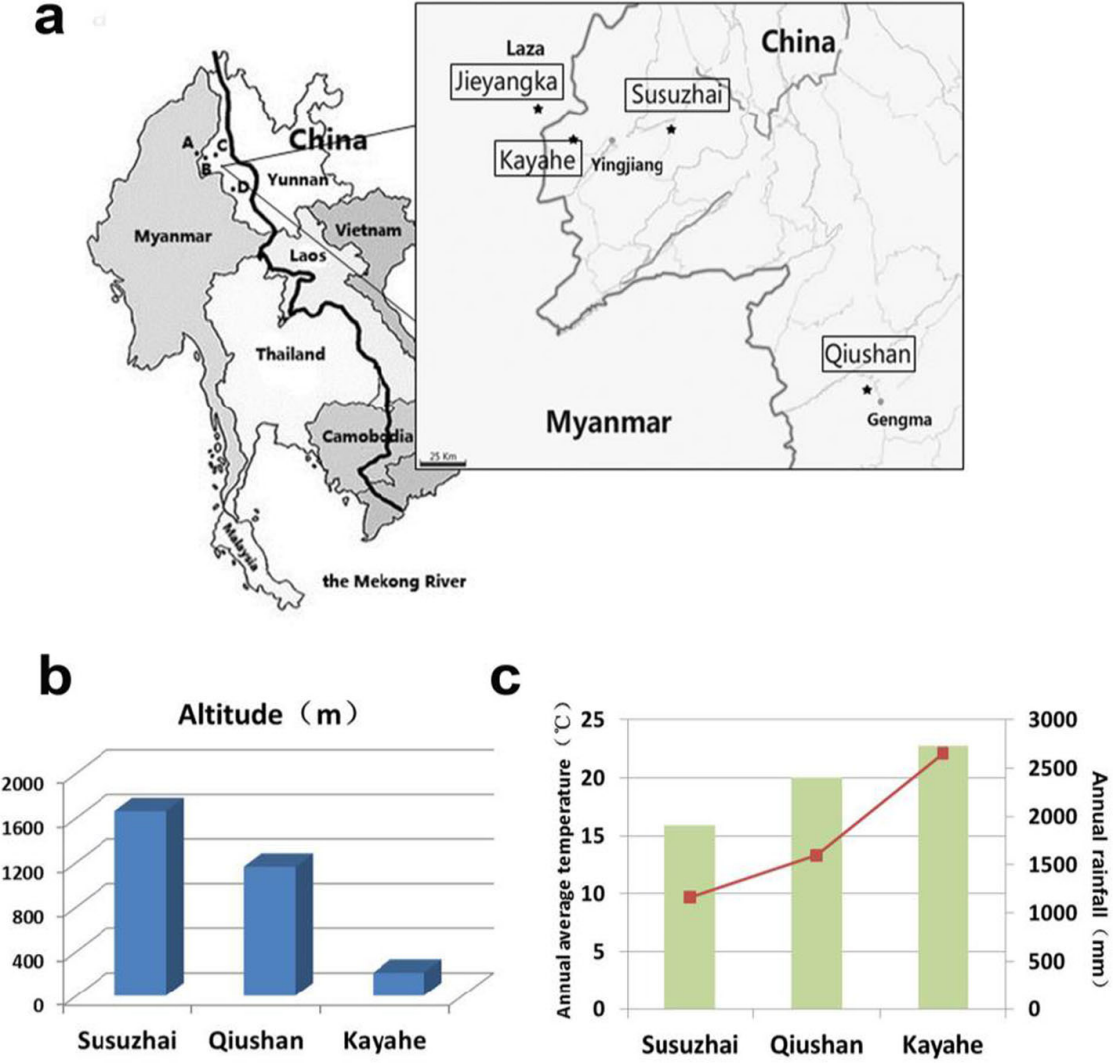
The information of research sites. **a** the specific locations of villages selected in this study. One village (Jieyangka) in Laza, Myanmar, three villages (Susuzhai, Qiushan and Kayahe) in Yunnan province, China. **b** the altitudes of three villages in Yunnan. **c** the annual average temperature and annual rainfall in the three villages selected in Yunnan

This study comprised two cross-sectional surveys conducted in the border area of China-Myanmar and Hainan province of China. The border area in Yunnan province with Myanmar [27, 28] was the most challenging area in which to achieve the final goal of a malaria-free China by 2020 as planned by the Chinese Center for Disease Control and Prevention. The malaria transmission intensity in these areas, especially *Pf* malaria, has decreased significantly in recent years because of the implementation of malaria control strategies. Only 15 indigenous *Pf* malaria cases were reported in Yunnan province in 2012–2013 [29]; thus, the sensitivity of traditional methods, such as EIR and parasite prevalence, is limited in this area. However, the number of indigenous *Pf* malaria cases was much greater in Laza, Myanmar, than Yunnan province in China; 415 *Pf* malaria cases were reported in 2012–2013 [30]. One village (Jieyangka) in Laza was selected; considering that malaria transmission is influenced by altitude, three villages at various altitudes in Yunnan were selected: Susuzhai (altitude 1 660 m), Qiushan (1 160 m), and Kayahe (210 m). The climates of these villages, such as temperature and rainfall, also vary (Fig. 1).

Hainan province used to be a malaria endemic area with the highest disease burden, but no locally acquired *Pf* malaria case has been reported since 2010 [31, 32]. In this study, we chose Danzhou of Hainan as a control for the historical *Pf* epidemic area.

### Design and participants

Cluster sampling was used in this study, and residents in all age groups were included from each village. Pregnant women were excluded from this study. All participants were requested to provide finger blood for immediate microscopy (thick and thin blood smears) and dried blood spots on filter paper (Whatman 3MM Chr) for subsequent serological tests. Two professional microscopists diagnosed all of the participants independently after reading the blood films. All of the blood filter papers were put in sealed plastic bags and stored at −80 °C after being dried at room temperature with abundant self-indicating silica desiccants. Information on gender and age was recorded.

### Non-epidemic controls

A total of 108 healthy volunteer donors from the General Hospital of Chinese People’s Liberation, Beijing, China, who had never been exposed to malaria were chosen as non-epidemic controls. Blood plasma and dried blood spots were prepared from each control. The plasma was used as a control for the plasma from malaria patients and the blood spots as a control for the dried blood spots collected from study sites.

### Ethical approval

Ethical approval was received from the Institutional Review Board (IRB) of the Institute of Basic Medical Sciences, Chinese Academy of Medical Sciences. Informed consent was obtained from all participants, and from guardians for children <15 years of age.

### Antigens and epitopes

M.RCAg-1 proteins were produced in *Escherichia coli* and prepared and purified by the Institute of Process Engineering, Chinese Academy of Science. The purity of the protein was verified through HP-SEC and RP-HPLC analysis and the stabilities examined by SDS-PAGE at regular intervals. All batches of proteins we obtained had good stability with purity >95 %. All epitopes were synthesized by China Peptides Co., Ltd, and the quality confirmed through mass spectrometry and HPLC analysis. The purity of all of the epitopes (from Epitope-1 to Epitope-11) included in the M.RCAg-1 protein was >95 %. All proteins and epitopes were split charged and stored at −80 °C.

### Elution of antibodies from dried blood spots

Antibodies were recovered from the blood spots following a propositional method [33]. Plastic bags containing blood spots were allowed to return to ambient temperature before opening and to check whether the silica desiccants remained blue. One disc approximately 3 mm in diameter was cut from each filter paper using a leather punch and placed in 1 000 μl of 3 % sheep serum dissolved in PBS–0.05 % Tween 20 (PBS-T). The solution was kept at 4 °C overnight with gentle mixing, and then used immediately for antibody detection or stored at −80 °C. The concentration of eluted serum proteins was equivalent to a 1:1000 dilution of the serum.

### Antibody assays

IgG antibodies to M.RCAg-1 protein and its epitopes were measured by indirect ELISA. Briefly, M.RCAg-1 was dissolved in 0.1 M Na_2_CO_3_ (pH 9.2) at 1 μg/ml (or 5 μg/ml for epitopes), coated on high absorbance plates (Corning), and kept at 4 °C overnight. After washing five times with PBST, the plates were blocked with 3 % (vol/vol) sheep serum in PBS following incubation for 2 h at 37 °C. Plasma diluted 1∶200 with blocking buffer or 100 μl of eluate from blood spots was added in duplicate and incubated and washed as described above. Next, the plates were incubated with peroxidase-conjugated goat anti-human IgG antibodies (Sigma) at a dilution of 1∶20 000. H_2_O_2_ with tetramethylbenzidine (TMB; Sigma) was chosen as the chromogenic substrate and the reaction terminated with 1 M H_2_SO_4_ after 10 min. The optical density (OD) was determined at both 450 and 630 nm using a microplate reader (Wellscan MK3, Labsystems Dragon, USA). Each plate contained three negative control serum samples from Beijing donors who had never been exposed to malaria and two positive control serum samples from malaria patients with high antimalarial antibody concentrations. All antibody values were expressed in arbitrary units (AU) calculated by dividing the mean OD by the mean OD + 3 standard deviations (SDs) for the three negative controls tested simultaneously. These relative OD values were referred to as OD %.

### Data processing and statistical analysis

Differences between the antibody concentrations of two groups were analyzed using the Mann-Whitney test. Spearman’s rank correlation test was used to test the relationship between antibody levels and different factors, including altitude, age, and parasitemia. The mean titer of the non-epidemic controls + 2 SDs was set as the cutoff and the antibody data dichotomized as seronegative or seropositive. A simple reversible catalytic model was fitted to the dichotomized data using maximum likelihood methods [34]. The model generates a seroconversion rate (SCR or λ) and a seroreversion rate (ρ). For this study, only the SCR was allowed to vary when models were fitted independently for each village; the seroreversion rate was fixed because independent reversion rates for each village did not improve the fit compared to using a common rate of reversion [16]. In each village, the 0–2 years age group was deleted because of distortions caused by the presence of maternal antibodies in highly endemic villages. Statistical analyses were carried out using IBM SPSS Statistics 19 for Windows and the above-mentioned models fitted using the Solver add-in in Excel (Microsoft Office, 2010).

## Results

### Study population

Details about the study population are given in Table 1. Fewer men were sampled than women in Jieyangka, but in Yunnan and Hainan the gender distribution was balanced. Samples from each village contained all age groups, except no children <5 years of age were sampled in Danzhou. The specific age distribution varied depending on the cluster, and the proportion of children under 5 years old was 27.3 % in Jieyangka, which is obviously higher than the proportions in other villages. A total of 15 *Pv* infections were detected in Jieyangka and 5 in Qiushan, Yunnan. No clinical symptom, such as fever, occurred. As no *Pf* infection was detected in the participants, it confirmed that the *Pf* malaria transmission intensity was low in all of these areas and the parasite prevalence not suitable for estimating the *Pf* malaria exposure level in these areas.

**Table 1:** The characteristics of study populations

		Myanmar	China			
		Laza	Yunnan			Hainan
		Jieyangka *n* = 589 (%)	Susuzhai *n* = 90 (%)	Qiushan *n* = 500 (%)	Kayahe *n* = 62 (%)	Danzhoun *n* = 150 (%)
Gender	Male	158(26.8)	44(48.4)	259(51.8)	24(38.7)	67(44.7)
	Female	431(73.2)	46(51.6)	241(48.2)	38(61.3)	83(55.3)
Age	<5	161(27.3)	8(8.9)	8(1.6)	8(12.9)	0(0)
	5–14	82(13.9)	12(13.3)	284(56.8)	12(19.4)	78(52)
	14–25	35(5.9)	27(30)	28(5.6)	3(4.8)	15(10)
	25–45	166(28.2)	20(22.2)	90(18)	18(29)	21(14)
	>45	145(24.6)	22(24.4)	91(18.2)	21(33.9)	36(24)
Infection	P. f	0	0	0	0	0
	P. v	15	5	0	0	0

#### Antibody response in serum from malaria patients

**Fig. 2:**
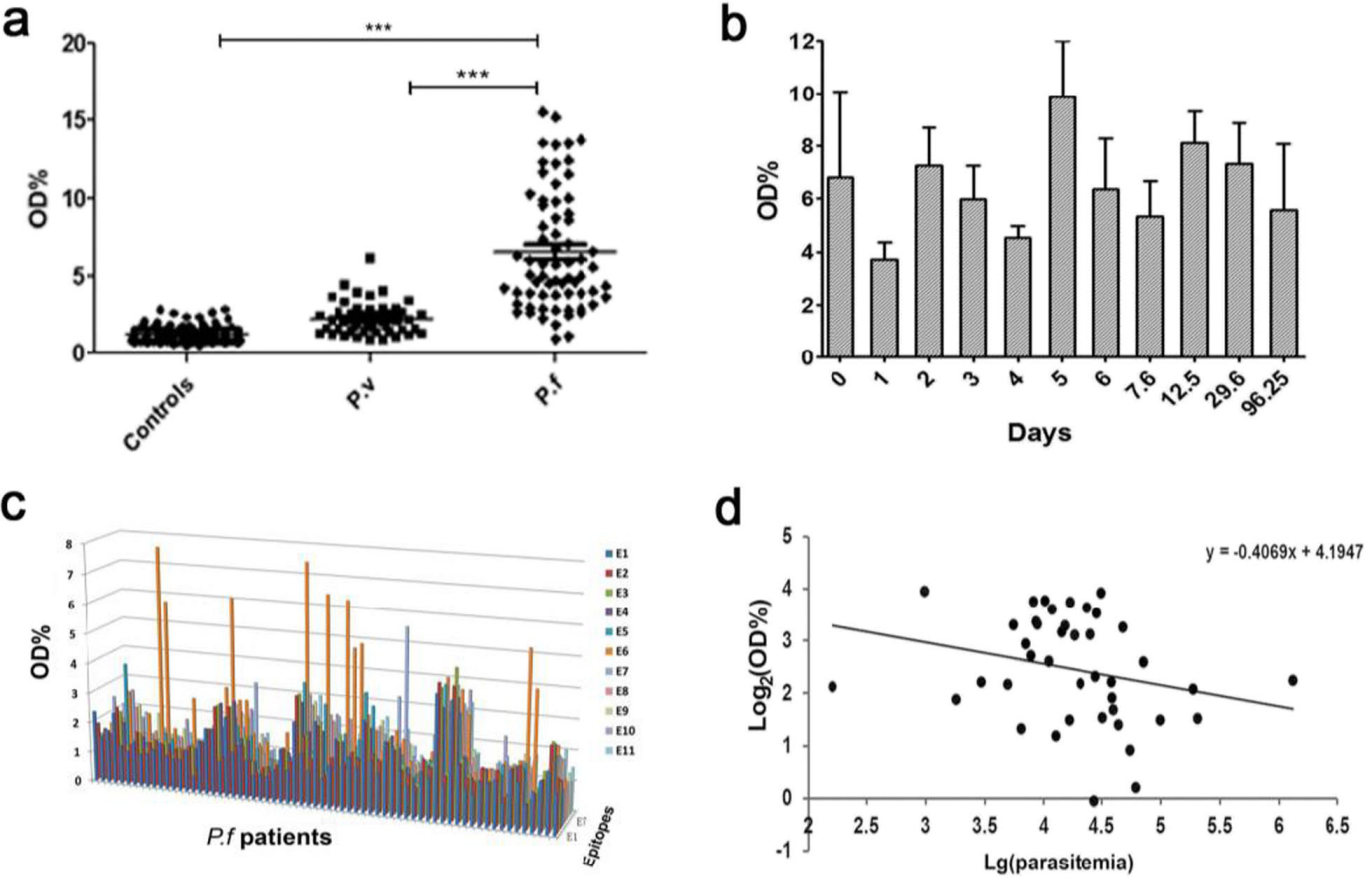
Antibody response to M.RCAg-1 and its epitopes in serums of malaria patients. **a** The IgG antibody levels against M.RCAg-1in *Pv, Pf* malaria patients and normal controls. **b** The X axis was days between the date of malaria onset and the date taking treatment, the Y axis showed the antibody levels. **c** Antibodies to all epitopes of M.RCAg-1. **d** The association between parasitemia and antibody levels

To investigate M.RCAg-1-specific antibody responses, we collected serum from malaria patients in Laza in 2008 before they took medicine (Fig. 2). The IgG antibody levels were predominantly higher in *Pf* patients than in *Pv* patients (*P* < 0.0001) and negative controls (*P* < 0.0001), and there was very limited cross-reactivity of M.RCAg-1 with *Pv* infection (Fig. 2a). The average antibody levels in *Pf* patients grouped by onset time are shown in Fig. 2b. These results suggest that anti-M.RCAg-1 antibody can be stimulated once malaria occurs and maintained at a certain concentration during the infection, though with some fluctuation. The levels of antibody to epitopes of M.RCAg-1 were also tested (Fig. 2c). All 11 epitopes could be identified to varying degrees by the naturally acquired antibodies in serum from *Pf* patients. A negative correlation was found between antibody levels and the parasitemia (*r* = -0.334, *P* < 0.05) in *Pf* patient serum (Fig. 2d), indicating that the anti- M.RCAg-1 antibodies offer immune protection against malaria.

#### Antibody variation after treatment

**Fig. 3:**
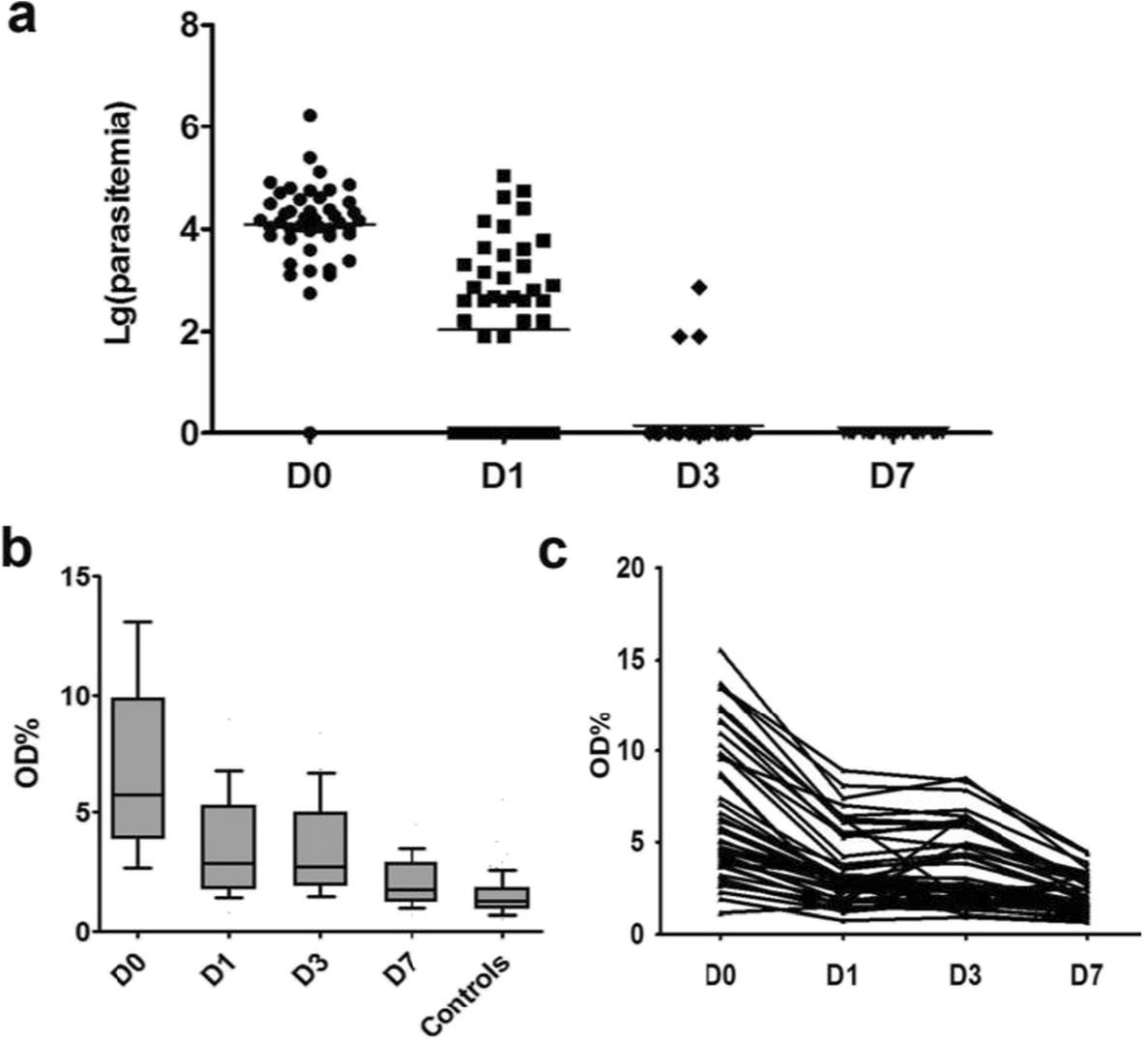
Antibody variation with reduction of parasitemia after treatment. **a** The parasitemia in different days after treatment. **b**The antibody levels of P. *falciparum* malaria patients in different days. **c** The antibody variation trends in each patient after treatment

To understand the longevity of anti-M.RCAg-1 antibody responses, we tested the antibody levels in serum samples taken from *Pf* patients at D0, D1, D3, and D7 (Fig. 3). The parasitemia decreased after taking medication (Fig. 3a), with an accompanying decrease in IgG antibodies, but the antibody levels at D7, when no parasites were detected, were still higher than in healthy controls from non-epidemic areas (*P* < 0.0001) (Fig. 3b). The longevity of anti-M.RCAg-1 antibodies was several weeks, as estimated by data from acutely infected individuals following drug treatment in this study. Furthermore, the trend in antibody variation in each patient presented good consistency (Fig. 3c), suggesting few polymorphisms in M.RCAg-1.

#### Antibody detection in blood spots

**Fig. 4:**
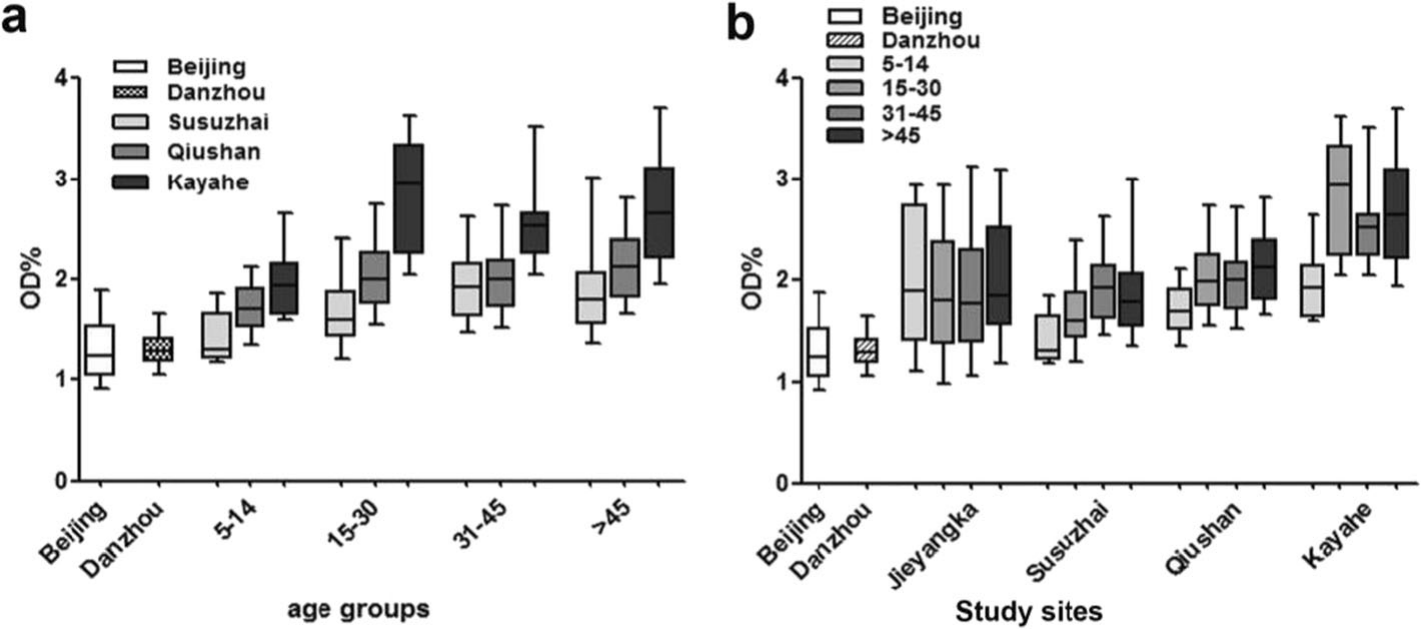
Anti-M.RCAg-1 antibody levels in different age groups and different study sites. **a** Grouped by age, to compare antibody levels of individuals from different study sites. **b** Grouped by study site, to compare antibody levels of individuals with different ages

To determine whether M.RCAg-1 can be used to estimate exposure to *Pf* malaria, especially in low transmission areas, we tested the antibody levels in eluates from blood spots collected from residents living in Hainan province and along the China-Myanmar border. We found no significant differences in the antibody levels of residents from Hainan and participants from Beijing (*P* = 0.176). However, on the China-Myanmar border, the antibody levels of residents from the four villages were significantly higher than the levels in participants from Beijing (*P* < 0.0001). In the three villages in Yunnan province, antibody levels decreased with increasing elevation (*r* = −0.258, *P* < 0.0001 in 5-14-year-olds; *r* = −0.492, *P* < 0.0001 in 15-30-year-olds; *r* = −0.356, *P* = 0.001 in 31-45-year-olds; and *r* = −0.473, *P* < 0.0001 in >45-year-olds; Fig. 4a). The antibody concentrations increased with increasing age (*r* = 0.421, *P* < 0.0001 in Jieyangka; *r* = 0.435, *P* < 0.0001 in Susuzhai; *r* = 0.451, *P* < 0.0001 in Qiushan; and *r* = 0.374, *P* = 0.003 in Kayahe; Fig. 4b). No difference in antibody levels was found between males and females for any of the villages (*P* > 0.05).

#### Evaluating malaria transmission intensity

**Fig. 5:**
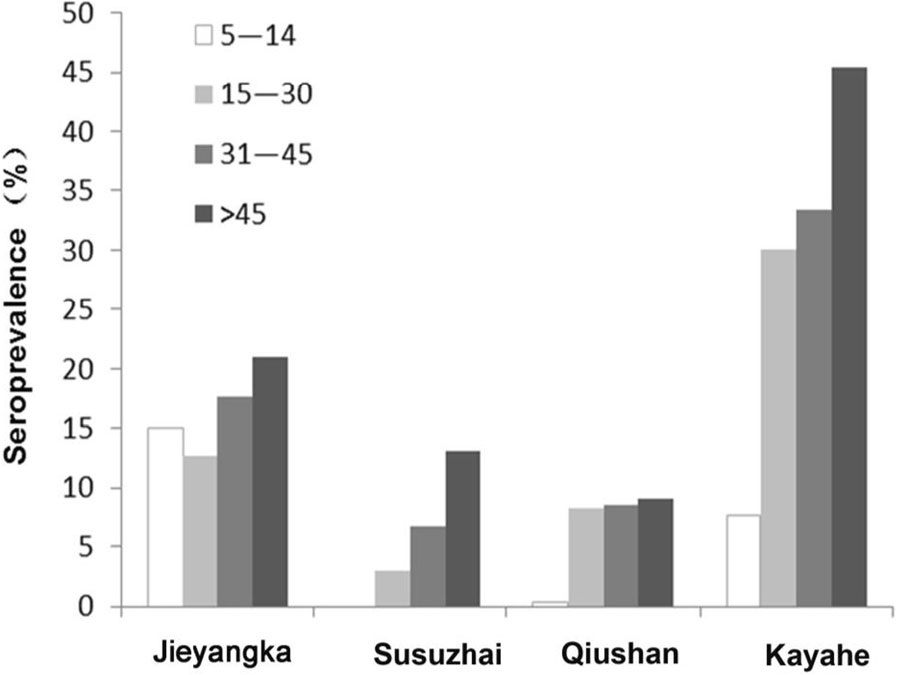
Seroprevalence in different age groups and different study sites

**Fig. 6:**
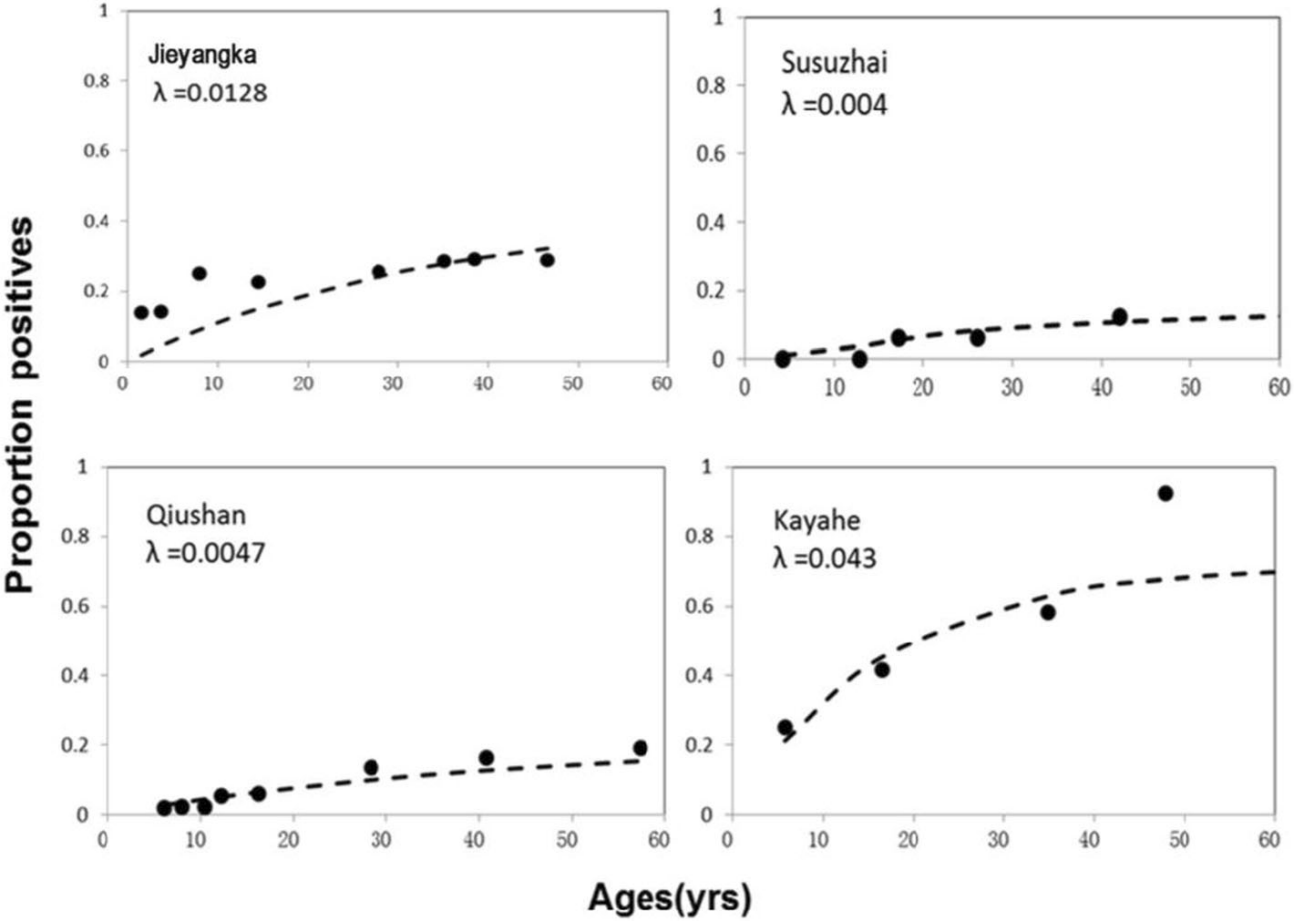
Age sero-prevalence plots for antibody responses to M.RCAg-1. The Maximum-likelihood fits of seroconversion (λ) from reversible catalytic equilibrium model for each village are acquired. Observed and estimated age-specific prevalence of antibodies represent with real points and dash line respectively

**Fig. 7:**
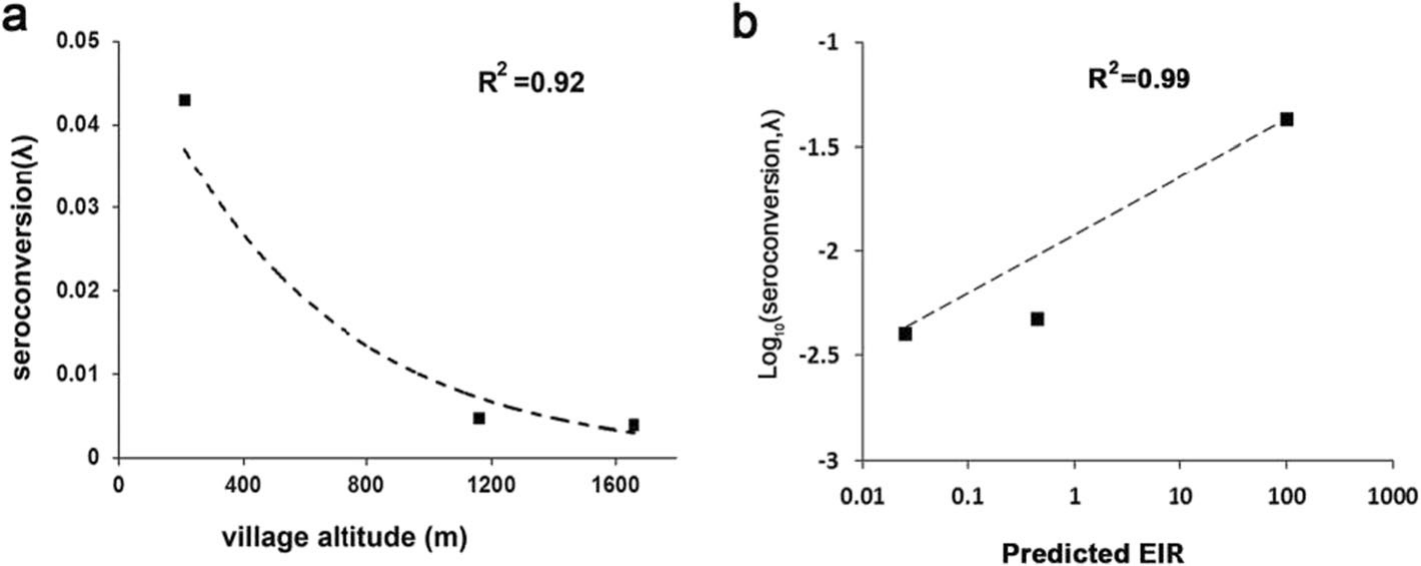
Association between altitude or EIR and rate of seroconversion from M.RCAg-1 seronegative to seropositive. **a** Plot of estimated seroconversion rates(λ) (calculated as for Fig. 6) against altitude. The line and R^2^ value are for the least-squares fitted functionλ = 0.0534e^-0.002h^, where h is the altitude in m. **b** Plot of log(λ) against log (predicted EIR). The least-squares fitted line has the equation Log_10_(λ) = 0.01*Log_10_(EIR)-2.3653. EIR was calculated from the equation log(EIR) = 2.523–0.0025 * altitude [35]

To obtain the parameter λ for each village, we calculated the seroprevalence data for each settlement stratified by age (Fig. 5), and then calculated the SCR using maximum-likelihood fits from a reversible catalytic equilibrium model (Fig. 6). Compared to the villages of Yunnan, the proportion of seroprevalence was higher in children <14 years old from Jieyangka, suggesting that the on-going *Pf* infection was more serious in this area. The parameter ρ was set to 0 in this study [5]. Parameter λ was 0.0128 for Jieyangka, 0.004 for Susuzhai, 0.0047 for Qiushan, and 0.043 for Kayahe. The age seroprevalence curve did not fit as well for Jieyangka as for the villages of Yunnan, showing that the observed seroprevalence in children <14 years of age was above the predicted curve. Theoretically, models should be fitted by two steps to calculate two forces of an infection profile when visual examination of the SCR suggests it is not uniform over the whole population, but finite-sized samples in this study limited further calculation. Therefore, the *Pf* malaria transmission in Jieyangka was underestimated here, especially in children under 14 years of age. Importantly, we identified a semi-logarithmic relationship between the village-specific rate of seroconversion (λ) and altitude, and the log (λ) linearly correlated with log (EIR), which was estimated from altitude [35] (Fig. 7).

## Discussion

Even though serological biomarker detection is theoretically the best tool for malaria surveillance, the properties of selected antigens are an important point of the technique. Only sensitive and specific antibodies can fulfill the requirements for estimating the malaria exposure level in a population precisely, monitor the changes over time and the impact of intervention on transmission, and confirm the elimination of malaria. The chimeric antigen M.RCAg-1 was identified using malaria parasite-immunized animal serum from a library containing thousands of chimeric antigen genes [36]. This antigen contains epitopes from eight *Pf* antigens that have been shown to play essential roles in malaria immune responses [15, 18, 37–39]. High antigenicity and immunogenicity has been demonstrated for M.RCAg-1 [24], and the antigenicity of individual epitopes included in the chimeric antigen has also been shown [40, 41].

Our data demonstrate that M.RCAg-1 can be recognized by serum from *Pf*-infected patients from Myanmar but not the serum of healthy individuals from several locations in China, which is in line with previous data from Brazil and Cameroon [24]. Our data also show that all epitopes included in M.RCAg-1 can be recognized by antibodies in the serum of *Pf*-infected patients, even if at different levels, suggesting that M.RCAg-1 is a well-recognized chimeric antigen to the naturally acquired anti-malaria antibodies and has the advantage of few polymorphisms. In addition, we found that anti-M.RCAg-1 antibodies rapidly appeared in the sera of patients when the infection started, confirming the sensitivity of M.RCAg-1 to *Pf* infection.

The specificity of sero-surveillance tests could be influenced by the potential cross-reactivity of antibodies to antigens from different malaria species [42–44]. Therefore, proper antigens must be either species-specific or have substantial sequence diversity between species. As our data show that anti-M.RCAg-1 antibody levels were significantly higher in *Pf*-infected patients than *Pv*-infected patients, we conclude that it is a *Pf*-specific sero-biomarker when used in malaria sero-surveillance.

One of the potential disadvantages of the serological approach is that, if antibody responses are very long-lived (such as MSP-1_19_), a serological assay may not distinguish significant recent deviations from the historic pattern of transmission [16]. Some studies have shown that, if not specifically focused on individuals with acute infection, investigators usually report longer half-lives than predicted from individuals with acute infection [45, 46]. In our study, we speculated that the longevity of the anti-M.RCAg-1 antibodies fell into the range of weeks to months, even with the addition of underestimated values caused by the research objects. These data show that M.RCAg-1 is suitable for estimating recent malaria transmission intensity.

In endemic areas, a decrease in malaria transmission will always wane the population immunity, which makes the local residents vulnerable to outbreaks of this disease. Therefore, immune monitoring is necessary for identifying the susceptible population and helping provide additional protective interventions, as demonstrated by Richards, who showed that sero-surveillance has the potential to indicate population immunity except exposure [47]. In this study, we observed that parasitemia decreases with the accumulation of anti-M.RCAg-1 antibodies before treatment, as has been reported in several prospective longitudinal studies in different parts of Africa and Asia [48, 49]. Anti-M.RCAg-1 antibodies have been suggested to have the potential to indicate anti-malaria immunity.

In our study, all of the areas selected for evaluating whether M.RCAg-1 can be used as a serological marker were areas where *Pf* malaria transmission have been reported to be dramatically decreased [50] and cannot be estimated using traditional methods, such as EIR and parasite prevalence. As our data showed that the anti-M.RCAg-1 antibody levels were significantly higher in participants from Yunnan and Laza than those from Beijing, the serum samples from epidemic areas are sensitive to this antigen, suggesting that M.RCAg-1 could be a proper sero-biomarker for estimating low malaria transmission intensity. In addition, because anti-M.RCAg-1 antibodies were not significantly different between residents from Hainan and Beijing, and considering that no local-acquired case of *Pf* infection has been reported in Hainan province since 2010, we conclude that M.RCAg-1 can be used to confirm the elimination of *Pf* malaria. Moreover, the anti-M.RCAg-1 antibody levels increased with the increasing age, which can be explained by the cumulative exposure to malaria parasites over time [51, 52].

Altitude has long been assumed to represent a proxy for malaria transmission [53] because there is a close relationship between temperature and rainfall, which plays important roles in the breeding of the malaria parasite carrier, mosquitos. In addition, Bodker’s group declared a highly significant decrease in the EIR with increasing altitude (log(EIR) = 2.523–0.0025 * altitude) [35]. The three villages we sampled close to the border of Yunnan province are under the same prevention and control system; therefore, the difference in malaria transmission intensity among them must be caused mainly by the altitude. Our study demonstrates that the estimated exposure rate (SCR) determined by M.RCAg-1 correlates with the traditional measure of transmission intensity (predicted EIR), providing great evidence for the use of M.RCAg-1 to estimate malaria transmission intensity.

From our studies, seropositivity is mainly due to adults in Yunnan province in China, whereas the seroprevalence is high in children under 14 years of age in Laza, Myanmar, meaning that the recent or on-going *Pf* malaria exposure in Laza is much more serious than in Yunnan. Our data are in line with a report on the malaria situation in the same areas [30] that mentions that varying socioeconomic status, medical conditions, and control measures for malaria make the differences between the two countries [30, 54].

Several reports have shown that using multiple antigens or epitopes in sero-surveillance assays has advantages over the use of one antigen [18, 47, 55]. Unlike other multi-antigen detectors using either antigen-coated beads or a mixture of several antigen proteins, M.RCAg-1 can be prepared from the supernatant of an *E. coli* expression system using a routine process. In addition, to test the serum antibodies with an indirect ELISA assay, all of these specialties make the task not only simple, but also inexpensive.

Many challenges still exist for malaria elimination as the focus shifts from the detection of symptomatic patients to the detection and clearance of all infections. Effective tests capable of estimating and monitoring transmission intensity with high sensitivity and accuracy in the field will play an important role. This study indicates that multi-epitope chimeric antigens may have a great advantage as serological markers to estimate *Pf* malaria transmission. However, more investigations in different populations are needed to confirm its usefulness and ensure the generalizability of results.

## Conclusions

Our study demonstrates that the chimeric multi-epitope antigen M.RCAg-1 has the potential as a sero-biomarker to estimate the *Pf* malaria transmission intensity, especially at a fairly low level, to monitor its recent trends, and to confirm the elimination of *Pf* malaria infection in malaria surveillance systems.

## Additional file

Additional file 1: Multilingual abstracts in the five official working languages of the United Nations. (PDF 958 kb)

## Abbreviations

EIR: Entomological inoculation rate
ELISA: Enzyme Linked Immunosorbent Assay
M.RCAg-1: Malaria Random Constructed Antigen-1
SCR: Seroconversion rate
