# Supplementary material for: Multi-epitope chimeric antigen used as a serological marker to estimate Plasmodium falciparum transmission intensity in the border area of China-Myanmar
Source: Infect Dis Poverty. 2016 Sep 7;5(1):98. doi: 10.1186/s40249-016-0194-x (PMC5015264; doi:10.1186/s40249-016-0194-x)
Supplement: Additional file 1: — Multilingual abstracts in the five official working languages of the United Nations. (PDF 958 kb) [file 40249_2016_194_MOESM1_ESM.pdf]

Translation of the abstract into the six official working languages of the United Nations

مستضد متعدد الحواتم خَيمَريٌّ مُستخدم كمؤشر مصلي لتقدير كثافة نقل المتصورة المنجلية في منطقة الحدود بين الصين وميانمار

مي شيويه ياو شياو دونج سون يو هوي جاو، تشى تشنج بن، وي وي دينج، جيا جيا تشانج، هنج وانج

ملخص

خلفية: في أعقاب انخفاض انتقال الملاريا في العديد من الدول والمناطق، أصبحت المعلمات المصلية مفيدة بشكل خاص لتقدير انتقال الملاريا في المناطق المنخفضة الحدة. هذه الدراسة تقييم علامة المصلية جديدة، المستضد-1 للملاريا عشوائي التركيب (M.RCAg-1)، والذي يحتوي على 11 من الحواتم من ثمانية مستضدات للمتصورة المنجلية، كأداة لتقييم كثافة انتقال الملاريا على طول منطقة الحدود بين الصين وميانمار.

الأسلوب: تم استخدام المصل من مرضى المتصورة المنجلية والمتصورة النشيطة للكشف عن خصائص M.RCAg-1 واستجابات الأجسام المضادة. أجريت عملية مسح مستعرضة في الحدود بين الصين وميانمار وفي مقاطعة هاينان في عام 2012 و 2013 باستخدام العينة العنقودية. جمعت أوراق ترشيح بقعة الدم من جميع المشاركين. تم الكشف عن الأجسام المضادة ضد M.RCAg-1 باستخدام الاليزا غير المباشر. تم إجراء اختبار مان ويتني واختبار معامل ارتباط الرتب لاسبيرمان لتحليل بيانات الأجسام المضادة. وقدرت كثافة انتقال ملاريا المتصورة المنجلية باستخدام نموذج التحويل التحفيزي على أساس احتمال الحد الأقصى لتوليد معدل التحول المصلي المجتمعي (SCR).

النتائج: تم التعرف بشكل جيد على M.RCAg-1 عن طريق الأجسام المضادة لمكافحة الملاريا المكتسبة طبيعيا في مرضى المتصورة المنجلية. وكان التفاعل المختلط محدودا جدا مع إصابة المتصورة النشيطة. وانخفض إجمالي كمية الأجسام المضادة مع انخفاض الطفيل بعد أخذ الدواء، واستمر عدة أسابيع. في مسح سكاني، كانت مستويات الأجسام المضادة أعلى في السكان الذين يعيشون بالقرب من الحدود بين الصين وميانمار من أولئك الذين يعيشون في مناطق غير الموبوءة (P <0.0001)، ولكن لم يلاحظ أي اختلاف كبير بين السكان في هاينان والمناطق غير الموبوءة. وكان التحول المصلي المجتمعي المحسوب 0.0128 لجيانجكا، 0.004 لسوسوجاي، 0.0047 لكويشان، و0.043 لكاياهي. كان معدل التعرض التقديري الناتج من مستوى الأجسام المضادة لمضادة M.RCAg-1 مرتبط مع المعايير التقليدية لكثافة الانتقال المستمدة من الارتفاع.

الخلاصة: تبين دراستنا أن M.RCAg-1 هو مفيد كمؤشر مصلي للتعرض للمتصورة المنجلية والملاريا، وخاصة بالنسبة للمراقبة الملاريا في مناطق العدوى المنخفضة.

Translated from English version into Arabic by Mahmoud Sami, through

TRANSLATORS WITHOUT BORDERS

应用多表位嵌合抗原评估中缅边境地区的恶性疟疾传播强度

姚美雪，孙晓东，高宇辉，程志斌，邓唯唯，张佳佳，王恒

摘要

引言：随着疟疾传播强度的降低，高灵敏性的血清学监测方法受到越来越多的重视，尤其对于评估传播强度较低的地区更为重要。本研究旨在评价多表位嵌合抗原 M.RCAg-1（含有来自 8 种恶性疟原虫特异性抗原的 11 个表位）用于评估恶性疟疾传播强度的有效性和可行性。

方法：阳性样本恶性疟和间日疟患者血清用于检测抗 M.RCAg-1 抗体反应的敏感性和特异性，疫区居民滤纸血片样本来自在中缅边境和中国海南的横断面调查取样。间接 ELISA 法用于抗体检测；秩和检验和秩相关分别用于差异性分析和相关性分析；使用 catalytic equilibrium model，结合二项分布和最大似然比方法，获得不同调查地点的恶性疟疾传播强度数据（血清阳转率，SCR）。

结果：M.RCAg-1 对恶性疟疾感染后产生的天然抗体敏感性强，且与间日疟疾感染没有交叉反应；人群多态性低；抗 M.RCAg-1 抗体在人体内半衰期较短。人群调查结果发现中缅边境疫区居民血清抗体水平高于北京非疫区对照

($P<0.0001$)；而海南省与北京无显著性差异。4 个村落的 SCR 值分别为：拉咱 0.0128，傈僳寨 0.004，邱山村 0.0047，卡牙河 0.043。利用 M.RCAg-1 获得的 SCR 与传统指标（昆虫接种率，EIR）存在线性相关。
结论：多表位嵌合抗原 M.RCAg-1 有用于疟疾疫区恶性疟疾传播强度评估的潜在价值，有可能被用于恶性疟疾消除阶段的监测工作。

Translated from English version into Chinese by Mei-Xue Yao

Antigène chimérique multi-épitopique utilisé à titre de marqueur sérologique pour l'estimation de l'intensité de transmission du *Plasmodium falciparum* dans la région transfrontalière sino-birmane

Mei-Xue Yao, Xiao-Dong Sun, Yu-Hui Gao, Zhi-Bin Cheng, Wei-Wei Deng, Jia-Jia Zhang, Heng Wang

Résumé
Contexte: à la suite de la diminution de la transmission du paludisme dans de nombreux pays et régions, des paramètres sérologiques sont devenus particulièrement utiles dans l'estimation de la transmission du paludisme dans les régions à faible intensité. La présente étude a évalué un nouveau marqueur sérologique, l'antigène-1 paludique à construction aléatoire (M.RCAg-1), contenant 11 épitopes de huit antigènes dirigés contre le *Plasmodium falciparum* et servant à évaluer l'intensité de la transmission du paludisme le long de la zone frontalière sino-birmane.
Méthode: du sérum provenant de patients infectés par le *P. falciparum* et le *Plasmodium vivax* a été utilisé pour détecter les propriétés du M.RCAg-1 et les réponses des anticorps. Des enquêtes transversales ont été menées à la frontière sino-birmane et dans la province du Hainan en 2012 et en 2013 à l'aide d'un échantillonnage en grappes. Des taches de sang sur papier filtre ont été collectées pour tous les participants. Des anticorps dirigés contre le M.RCAg-1 ont été utilisés dans le cadre d'un test ELISA indirect. Le test de Mann-Whitney et le test de corrélation de Spearman ont été réalisés afin d'analyser les données relatives aux anticorps. L'intensité de la transmission du paludisme à *P. falciparum* a été estimée à l'aide d'un modèle de conversion catalytique sur la base de la probabilité maximale de génération d'un taux de séroconversion communautaire (SCR).
Résultats: le M.RCAg-1 a bien été reconnu par les anticorps antipaludiques naturellement acquis chez les patients infectés par le *P. falciparum* et ne présentait qu'une réactivité croisée très limitée avec l'infection à *P. vivax*. La quantité totale des anticorps IgG a diminué simultanément à la baisse de la parasitémie suite à la prise de médicaments et cette réduction a duré plusieurs semaines. Dans le cadre d'une enquête menée auprès de la population, les concentrations d'anticorps étaient plus élevées chez des personnes vivant à proximité de la frontière sino-birmane par rapport aux personnes vivant dans des zones non épidémiques ($P<0,0001$), mais aucune différence significative n'a néanmoins été observée parmi les résidents du Hainan et de zones non épidémiques. Le SCR calculé s'élevait à 0,0128 pour Jieyangka, 0,004 pour Susuzhai, 0,0047 pour Qiushan, et 0,043 pour Kayahe. Le taux d'exposition estimé et obtenu à partir du taux d'anticorps anti-M.RCAg-1 était corrélé à des mesures traditionnelles de l'intensité de la transmission dérivées de l'altitude.
Conclusion: notre étude démontre que le M.RCAg-1 est potentiellement utile en tant qu'indicateur sérologique d'une exposition au paludisme à *P. falciparum*, notamment pour la surveillance du paludisme dans les zones à faible taux de transmission.

Translated from English version into French by eric ragu, through

TRANSLATORS
WITHOUT BORDERS

Используется мульти-эпитоп химерный антиген в качестве серологического маркера для оценки интенсивности передачи малярийного плазмодия в пограничном районе Китая и Мьянмы

Mei-Xue Yao, Xiao-Dong Sun, Yu-Hui Gao, Zhi-Bin Cheng, Wei-Wei Deng, Jia-Jia Zhang, Heng Wang

Реферат

Фон: Со снижением передачи малярии во многих странах и регионах, серологические параметры становятся особенно полезным для оценки передачи малярии в районах с низкой интенсивностью. Это исследование оценило мульти-эпитоп химерный антиген M.RCAg-1, который содержит 11 эпитоп из восьми малярийного плазмодия антигена, в качестве инструмента для оценки интенсивности передачи малярии вдоль пограничной зоны Китая и Мьянмы.

Метод: Сыворотка P. Falciparum и P. Vivax тропической малярии и плазмодий трехдневной пациентов для обнаружения свойств M.RCAg-1 и реакции антител. Поперечные исследования были проведены на границе Китая и Мьянмы и в провинции Хайнань. Фильтр крови пятна бумаги были собраны из всех участников. Антитела против M.RCAg-1 были обнаружены с помощью способа непрямого ELISA. Манна-Уитни и тест ранговой корреляции были проведены для анализа данных антител. Интенсивность передачи трехдневной малярии П. оценивали с использованием каталитической модели преобразования на основе максимального правдоподобия генерации скорости сообщества сероконверсии (SCR).

Результаты: У M.RCAg-1 есть естественные антитела со сильной чувствительностью, которые возникаются после тропической малярийной инфекции, кроме этого, у него с трехдневной малярийной инфекции нет перекрестной реактивности; низкий полиморфизм популяции; антитело анти M.RCAg-1 имеет более короткий период полураспада в организме человека. Опрос населения показало, что Сывороточный уровень антител жители заражённого района на Китайско-Бирманской границе выше, чем уровень жителей незаражённого района Пекина (p <0,0001); и между Хайнан и Пекин не было существенной разницы. Значения SCR четырех деревень: Laiza 0,0128, 0,004 Лису Село, деревня Цю Шаня 0,0047, 0,043 Ка-я Река. Между значением SCR по методу M.RCAg-1 с традиционными параметрами (насекомое скорость прививка, EIR) существует линейная корреляция.

Вывод: мульти-эпитоп химерный антиген M.RCAg-1 имеет потенциальную ценность для оценки интенсивности распространения тропической малярии заражённых малярии районах, она может быть использована для контрольной работы на этапе устранении тропической малярии.

Translated from English version into Russian by Fo-Bin Xie

Uso de un antígeno quimérico multiepítope como marcador serológico para estimar la intensidad de transmisión de *Plasmodium falciparum* en la zona de la frontera entre China y Myanmar

Mei-Xue Yao, Xiao-Dong Sun, Yu-Hui Gao, Zhi-Bin Cheng, Wei-Wei Deng, Jia-Jia Zhang, Heng Wang

Resumen

Antecedentes: Tras la disminución de la transmisión de la malaria en muchos países y regiones, los parámetros serológicos han pasado a ser especialmente útiles para estimar la transmisión de la malaria en zonas de baja intensidad. Este estudio evaluó un nuevo marcador serológico, antígeno aleatorio de la malaria-1 (M.RCAg-1), que contiene 11 epítopes de ocho antígenos de *Plasmodium falciparum*, como herramienta para evaluar la intensidad de la transmisión de la malaria en la zona de la frontera entre China y Myanmar.

Método: Se utilizó el suero de pacientes con *P. falciparum* y *Plasmodium vivax* para detectar las propiedades del M.RCAg-1 y las respuestas de los anticuerpos. Se llevaron a cabo encuestas transversales mediante el uso de muestreo por conglomerados en la frontera entre China y Myanmar y en la provincia de Hainan en 2012 y 2013. Se recolectaron papeles absorbentes de todos los participantes. Se detectaron anticuerpos contra el M.RCAg-1 mediante el uso de ELISA indirecta. Para analizar la información de los anticuerpos se realizaron pruebas de Mann-Whitney y de coeficiente de correlación por rangos de Spearman. La intensidad de transmisión de la malaria por *P. falciparum* se estimó mediante el uso de un modelo de conversión catalítica basado en la máxima probabilidad de generación de una tasa de seroconversión comunitaria (SCR).

Resultados: El M.RCAg-1 fue bien reconocido por los anticuerpos naturalmente adquiridos contra la malaria en pacientes con *P. falciparum* y tuvo reactividad cruzada muy limitada con la infección por *P. vivax*. Lacantidad total de anticuerpos IgG disminuía con la disminución de la parasitemia luego de tomar medicación y duró varias semanas. En una encuesta de la población, los niveles de anticuerpos fueron más elevados en los residentes que vivían cerca de la frontera entre China y Myanmar que en aquellos que vivían en zonas no epidémicas ($P<0.0001$), pero no se observó una diferencia significativa entre los residentes de Hainan y las zonas no epidémicas. La SCR calculada fue de 0,0128 para Jieyangka, 0,004 para Susuzhai, 0,0047 para Qiushan y 0,043 para Kayahe. La tasa de exposición obtenida del nivel de anticuerpos anti M.RCAg-1 tenía relación con las medidas tradicionales de intensidad de transmisión derivadas de altura.

Conclusión: Nuestro estudio demuestra que el M.RCAg-1 es potencialmente útil como indicador serológico de la exposición a la malaria por *P. falciparum*, en particular para el monitoreo de malaria en zonas de baja transmisión.

Translated from English version into Spanish by Maria Alejandra Aguada, through

TRANSLATORS
WITHOUT BORDERS
